# Coeliac disease is associated with depression in children and young adults with type 1 diabetes: results from a multicentre diabetes registry

**DOI:** 10.1007/s00592-020-01649-8

**Published:** 2021-01-23

**Authors:** Sascha René Tittel, Désirée Dunstheimer, Dörte Hilgard, Burkhild Knauth, Elke Fröhlich-Reiterer, Angela Galler, Michael Wurm, Reinhard Walter Holl, For the DPV Initiative

**Affiliations:** 1 Institute for Epidemiology and Medical Biometry, ZIBMT, Ulm University, Albert-Einstein-Allee 41, 89081 Ulm, Germany; 2 German Center for Diabetes Research (DZD), Munich-Neuherberg, Germany; 3 Paediatrics and Adolescent Medicine, Medical Faculty University of Augsburg, Augsburg, Germany; 4 Department of Pediatrics, Witten, Germany; 5 Department of Pediatrics and Adolescent Medicine, CJD Berchtesgaden, Berchtesgaden, Germany; 6 Division of General Pediatrics, Department of Paediatrics and Adolescent Medicine, Medical University Graz, Graz, Austria; 7 Charité - Universitätsmedizin Berlin, corporate member of Freie Universität Berlin, Humboldt-Universität zu Berlin, and Berlin Institute of Health, Berlin, Germany; 8 Clinic St. Hedwig, University Children’s Hospital Regensburg (KUNO Clinics), University of Regensburg, Regensburg, Germany

**Keywords:** Coeliac disease, Depression, Paediatric, Endocrinology, DPV

## Abstract

**Aims:**

To analyse the association between coeliac disease (CD) and depression in children, adolescents, and young adults with type 1 diabetes (T1D).

**Methods:**

We included 79,067 T1D patients aged 6–20 years, with at least six months of diabetes duration, and treatment data between 1995 and 2019 were documented in the diabetes patient follow-up registry. We categorized patients into four groups: T1D only (*n *= 73,699), T1 + CD (*n *= 3379), T1D + depression (*n *= 1877), or T1D + CD + depression (*n *= 112).

**Results:**

CD and depression were significantly associated (adjusted OR: 1.25 [1.03–1.53]). Females were more frequent in both the depression and the CD group compared with the T1D only group. Insulin pumps were used more frequently in T1D + CD and T1D + depression compared with T1D only (both *p *< .001). HbA1c was higher in T1D + depression (9.0% [8.9–9.0]), T1D + CD + depression (8.9% [8.6–9.2]), both compared with T1D only (8.2% [8.2–8.2], all *p *< .001). We found comorbid autism, attention deficit hyperactivity disorder, anxiety, schizophrenia, and eating disorders more frequently in the T1D + CD + depression group compared with T1D only (all *p *< .001).

**Conclusions:**

CD and depression are associated in young T1D patients. The double load of T1D and CD may lead to an increased risk for depression. Depression was associated with additional psychological and neurological comorbidities. Aside from imperative CD screening after T1D diagnosis and regular intervals, depression screening might be helpful in routine care, especially in patients with diagnosed CD.

**Supplementary Information:**

The online version contains supplementary material available at (10.1007/s00592-020-01649-8).

## Introduction

Coeliac disease (CD) is an autoimmune-mediated disorder resulting in intestinal damage and mucosal atrophy on consumption of gluten [1]. CD is a common comorbidity in type 1 diabetes (T1D) with an average prevalence of approx. 5% versus 1% in the general population [2]. In an international comparison of children and adolescents with T1D, CD prevalence has been reported to range between 1.6% and 16.4% [3]. Prevalence of CD is increasing, even in regions like Asia, where gluten is now consumed more frequently due to changes in eating habits and infant feeding [4].

The first description of CD was given by Samuel Gee in 1888, suggesting dietary treatment; however, wheat, rye, and oats as toxic components were not identified until 1950 [5]. Earlier publications discussing CD and behavioural abnormalities presented case reports of phlegmatic, “difficult,” or “naughty” infants and children with overall bad behaviour, crying attacks of unknown reasons during the night, low appetite, and general negativity [6, 7], which improved after initiation of a gluten-free diet (GFD).

Guidelines by the European Society for the Study of Coeliac Disease recommend duodenal biopsies for CD diagnosis in adults [8], especially if patients have pre-existing T1D, family history of CD, or other associated comorbidities [9]. CD in adults is often associated with concomitant extra-intestinal symptoms including anxiety, depression, thoughts of self-harm, dementia, epilepsy, attention deficit hyperactivity disorder (ADHD), autism, eating disorders, infertility, and others [10–17].

However, not every patient with CD experiences symptoms [18]. As a genetic component of CD has been recognized, family members of CD patients are often screened before symptoms occur [19, 20]. The recent ESPGHAN guidelines postulate antibody testing in all suspected CD cases with subsequent biopsy in case the antibody titers are not more than 10 times the upper limit of the reference range [1]. Based on the increased prevalence of CD in T1D, the ISPAD guidelines also recommend CD screening at diagnosis of T1D and at 2 and 5 years thereafter [17]. The multinational TEDDY study focused on genetic and environmental risk factors for T1D and CD in newborns and infants that showed typical HLA haplotypes [21].

Until today, the only way to treat CD and prevent symptoms is via strict GFD, since even the smallest amounts of gluten can cause the onset or aggravation of CD symptoms [12]. By diagnosing CD and strictly adhering to a GFD, the occurrence of symptoms can be prevented.

According to the World Health Organization (WHO), the global prevalence of depressive disorders in children and adolescents ranges from 3–4.5% [22]. In young T1D patients, the prevalence of depression ranges from 15 to 30% [23–25].

The treatment of T1D, CD, and depression all requires different knowledge in the respective treatment and therefore a multidisciplinary team. To our knowledge, there are no studies about the combined occurrence of depression and CD in paediatric and young adult T1D patients. Our aim is to fill that gap and compare young T1D patients with CD and/or depression to T1D patients with neither CD nor depression, investigating demographic and therapy-related outcomes. In addition, we assessed possible associations with further psychological, neurological, and microvascular comorbidities.

## Materials and methods

### Participants

We included patients from the diabetes prospective follow-up registry (DPV) that fulfilled the following criteria: (1) T1D diabetes onset at age ≥ 6 months, (2) age 6–20 years, (3) at least 6 months of diabetes duration, and (4) treatment data available between January 1995 and December 2019.

DPV is a multicentre initiative comprising 588,860 diabetes patients (March 2020) and 501 centres in Germany, Austria, Switzerland, and Luxembourg. In total, 476 centres contributed data to this analysis. More than 90% of paediatric T1D patients in Germany are estimated to be documented in the DPV [2]. Data collection and analysis of anonymized data from the DPV registry were approved by the Medical Faculty Ethics Committee of Ulm University, Germany, and by the local review boards of participating centres. Semi-annually, centres send pseudonymized data to Ulm, Germany, where data are validated, anonymized, and added to the cumulative database [2].

We assigned patients to mutually exclusive groups: T1D only, T1D + CD, T1D + depression, or T1D + CD + depression based on all observed data from that patient in the DPV. T1D only was our reference group.

### Variables

CD diagnosis was based on clinical diagnosis (in part based on antibody results) by the treatment centre, with or without confirmation by small bowel biopsy. Depression was defined by clinical diagnosis (ICD10 and DSM-V) or specific pharmacotherapy [26]. Demographic data were age, diabetes duration, age at diabetes diagnosis, sex, and migration background (at least one parent or patient not born in Germany, Austria, Switzerland, or Luxembourg). Outcomes of interest were BMI-SDS, HbA1c, type of insulin therapy (injection vs. pump therapy), severe hypoglycaemia, diabetic ketoacidosis (DKA), and psychological/neurological comorbidities including autism, ADHD, schizophrenia, anxieties, and eating disorders (anorexia, bulimia, binge eating, others). Psychological and neurological comorbidities were documented by appropriate diagnosis terms or ICD10 codes by experienced psychologists that are present in every certified diabetes centres participating in DPV. We also analysed microalbuminuria (defined by urine albumin-to-creatinine ratio ≥ 30 mg/g) and retinopathy. Patients with missing information on retinopathy or nephropathy were excluded from the respective analysis only. HbA1c was standardized to the reference range of 4.05%—6.05% according to the Diabetes Control and Complication Trial (DCCT) using the multiple of the mean method [2]. For BMI-SDS values, we used AGA (German paediatric obesity working group) reference data [27]. We defined severe hypoglycaemia as hypoglycaemic episode with cognitive impairment (with or without coma) necessitating help from another person [2]. DKA was defined as pH < 7.3 and/or serum bicarbonate < 15 mmol/l.

### Data aggregation

We aggregated data as medians for all records during the most recent treatment year of each patient. Depression, CD, and psychological comorbidities were aggregated per lifetime.

### Statistical methods

In the unadjusted analysis of demographic and clinical variables (Table 1), medians and interquartile ranges (IQR) are presented for continuous variables and percentages for categorical variables. We used Wilcoxon’s rank-sum test for unadjusted group comparisons of continuous variables and ${\chi }^{2}$-test for categorical variables. Two-sided p-values were adjusted for multiple testing according to Bonferroni–Holm, and significance was set at < 0.05. For HbA1c and BMI-SDS, we used linear regression models and reported outcomes as adjusted least-square means [95% confidence interval (CI)], and logistic models for the association of CD with depression (odds ratio with 95% CI), and the frequency of other comorbidities (least-square mean with 95% CI). Severe hypoglycaemia or DKA rates per patient-year (least-square mean with 95% CI) were calculated using negative binomial regression models with individual time under risk as offset. All regression models were adjusted for sex, age group, age at diabetes onset group, and migration background. Age was categorized as 6–12, > 12–18, > 18–20 years and age at diabetes onset as ≤ / > 10 years. For sensitivity analysis, we adjusted the microalbuminuria and retinopathy models additionally for HbA1c groups (≤ / > 7.5% (58.5 mmol/mol)).

**Table 1:** Characteristics of T1D patients during most recent treatment year

	Total	T1D + CD + D	T1D + CD	T1D + D	T1D only	*p*-value T1D only versus T1D + CD + D	*p*-value T1D only versus T1D + CD	*p*-value T1D only versus T1D + D
Number of cases	79,067	112	3379	1877	73,699			
Male sex [%]	52.8	34.8	44.3	42.7	53.5	< .001	< .001	< .001
Age [y]	16.5 [13.3–17.8]	17.3 [15.6–18.9]	15.6 [11.9–17.6]	17.4 [16.1–18.5]	16.5 [13.3–17.8]	< .001	< .001	< .001
Diabetes duration [y]	6.0 [3.2–9.4]	8.9 [5.5 –12.4]	7.3 [4.3–11.2]	6.8 [4.1–10.4]	5.9 [3.1–9.3]			
Age at diabetes diagnosis [y]	8.9 [5.5–12.1]	7.7 [4.0–11.4]	6.2 [3.4–9.8]	10.1 [6.7–12.7]	9.0 [5.6–12.1]	.11	< .001	< .001
Pump therapy [%]	39.1	48.6	53.3	42.7	38.3	.11	< .001	< .001
Severe hypoglycaemia [%]	7.2	8.0	5.7	9.1	7.2	.73	< .01	< .01
Diabetic ketoacidosis [%]	2.4	6.3	1.8	6.5	2.3	.03	.07	< .001
Migration background [%]	17.4	24.1	18.9	17.8	17.3	.12	.03	.60

Data are presented as median [interquartile range] or as percentage

*p*-values were adjusted for multiple testing

*CD* Coeliac disease, *D* depression, *T1D* type 1 diabetes

## Results

Among 79,067 T1D patients, 1,877 (2.4%) had comorbid depression, 3,379 (4.3%) had CD (62.2% biopsy-proven), and 112 (0.1%) patients had CD and depression (65.2% biopsy-proven). T1D only was found in 73,699 (93.2%). We found a significant association between CD and depression (adjusted OR: 1.25 [1.03–1.53]). For sensitivity analysis, we calculated the same model with biopsy-proven CD only, where the association was not significant (OR: 1.26 [0.99–1.60]).

Patients with T1D + CD were younger, and patients with T1D + depression (with or without additional CD) were older compared to the reference group (all *p* < 0.001, Table 1). We found an imbalance regarding sex distribution in all comorbidity groups. (Females were more common in the three CD/depression groups compared with the reference group (all *p *< 0.001).) There were no significant differences in the proportions of patients with migration background between the groups, except in T1D + CD compared with T1D only (18.9% vs. 17.3%, *p *= 0.03). Pump usage was more frequent in T1D + CD and in T1D + depression patients (both *p *< 0.001) compared with the reference group (Table 1).

**Fig. 1:**
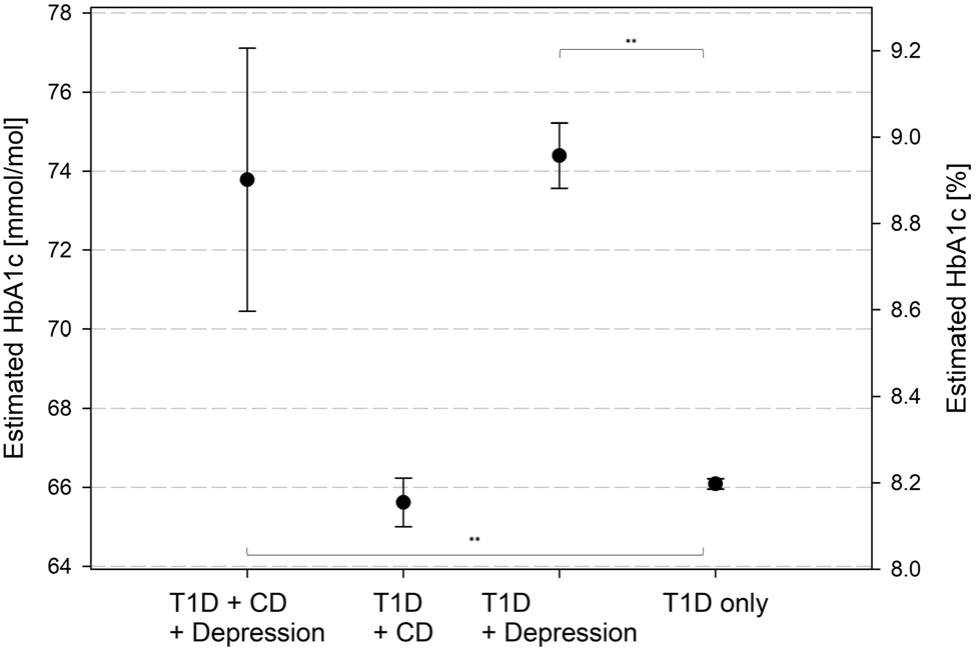
Estimated HbA1c adjusted for age, age at diabetes onset, sex, migration background, treatment year. T1D: type 1 diabetes; CD: coeliac disease. *p-*values: ** < .001 95% confidence intervals

**Fig. 2:**
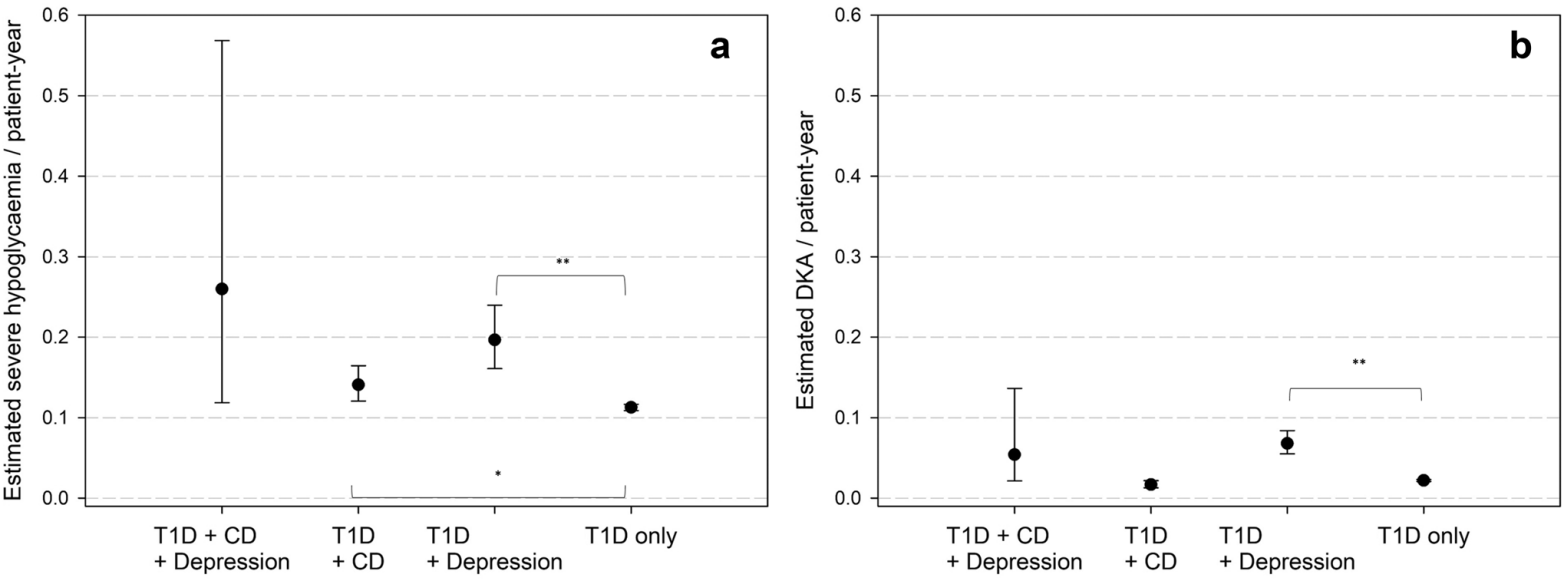
Estimated event rates for glycaemic decompensation adjusted for age, age at diabetes onset, sex, migration background, treatment year. T1D: type 1 diabetes; CD: coeliac disease. a: severe hypoglycaemia, b: diabetic ketoacidosis (DKA). *p*-values: * < .05; ** < .001

T1D + CD patients had a lower adjusted BMI-SDS (0.36 [0.33–0.40]) compared with T1D only (0.52 [0.51–0.53]) and T1D + depression patients (0.55 [0.51–0.60], all *p *< 0.001). There was no difference between T1D + CD and T1D + CD + depression in BMI-SDS. T1D + depression with or without CD was associated with worse metabolic control compared with T1D only (both *p *< 0.001, Fig. 1). We found higher rates of DKA in T1D + depression patients compared to the reference group and higher rates of severe hypoglycaemia in T1D + depression and T1D + CD compared with the reference group (Fig. 2).

**Fig. 3:**
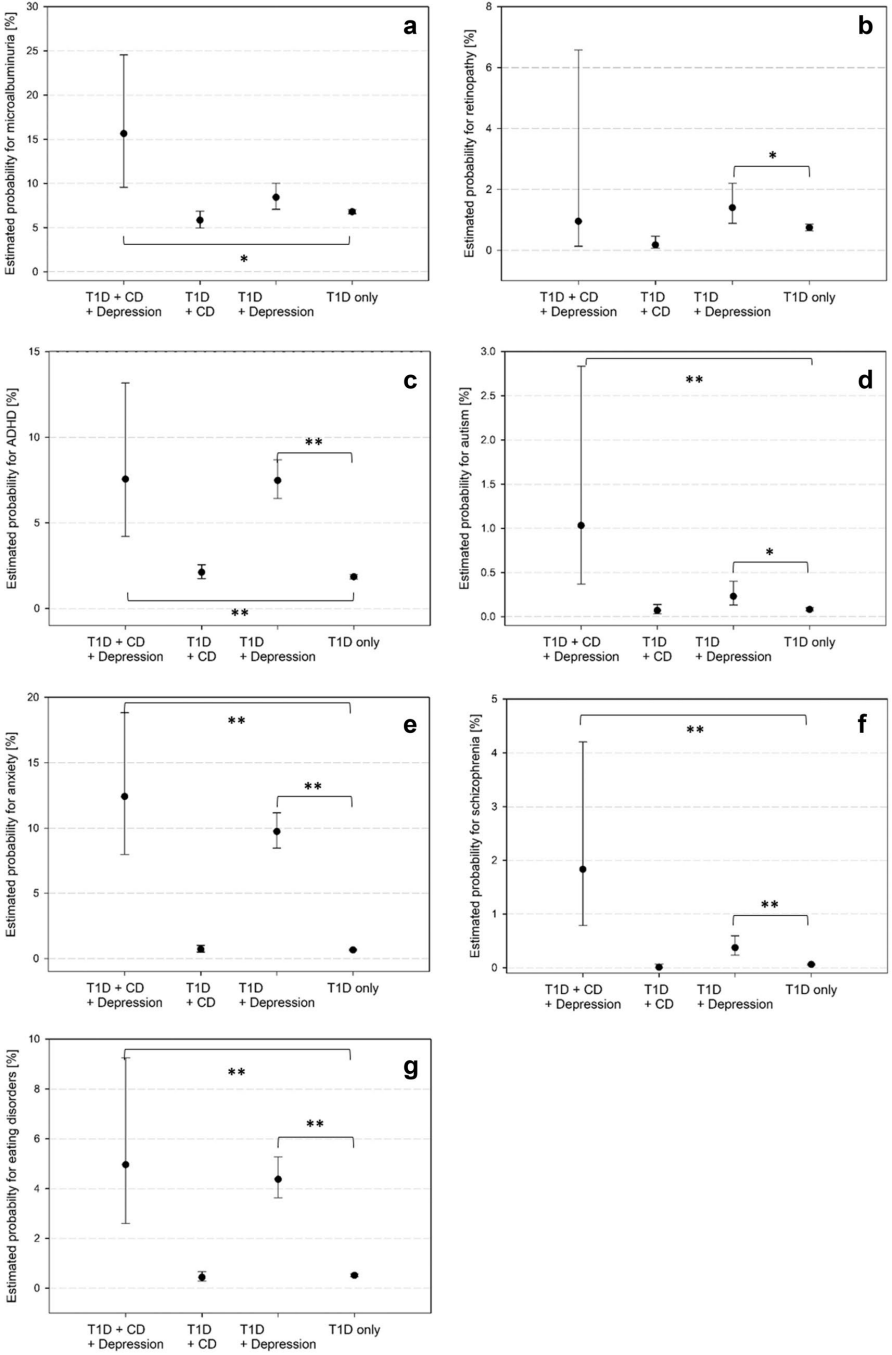
Probabilities for comorbidities were calculated via logistic regression and adjusted for age, age at diabetes onset, sex, migration background, treatment year. T1D: type 1 diabetes; CD: coeliac disease. a: Microalbuminuria, b: Retinopathy, c: ADHD, d: Autism, e: Anxiety disorders, f: Schizophrenia, g: Eating disorders. *p*-values: * < .05; ** < .001

Microalbuminuria was more frequent in patients with T1D + CD + depression (Fig. 3a). Adjusting for HbA1c did not have an impact on this result. Retinopathy occurred more often in T1D + depression compared with the reference group, even after HbA1c adjustment (Fig. 3b). In patients with T1D + depression with or without CD, we found higher rates of autism or ADHD (Fig. 3c, d), as well as anxiety, schizophrenia, or eating disorders (Fig. 3e, f, g) compared with the reference group.

## Discussion

We found a positive association between CD and depression among children and young adults with T1D. In the general population, CD is associated with psychological disorders, such as depression [12], with a lifetime prevalence of up to 31% in CD patients compared with 7% of patients without CD [28]. Our study confirms this association for usually asymptomatic CD in patients with T1D. Causes might include excessive cytokine secretion, or iron deficiency from malabsorption, reducing dopamine levels [12, 29, 30]. Early detection of CD is important, since adhering to a GFD may alleviate depressive symptoms [12, 31, 32]. Having T1D and additional CD is a heavier burden than having only one chronic disease. If we assume that depression develops later than CD, it is not surprising that the double load may lead to an increased risk for depression. However, we cannot say whether the increased risk for depression in CD is due to a restricted diet or due to untreated CD.

In particular, young patients with CD without T1D may experience exclusion from everyday life or bullying due to being different or the special diet they have to adhere to [33, 34]. Psychosocial distress may also derive from costs or availability of gluten-free food [35]. Since lifelong adherence to a GFD is a difficult task, it is to argue whether adhering to a strict diet in patients with only light symptoms and positive CD antibodies is preferable, or whether this has negative psychological effects. There exist conflicting data on a possible benefit of GFD in patients with only light or no symptoms [36, 37]. While there are reports of improved quality of life after GFD initiation, some report negative feelings in patients with GFD and reduced pleasure concerning meals, and even higher rates of depression [12, 38].

Eating disorders have a known association with T1D as well as with CD and depression [12, 39]. Comorbid eating disorders or incomplete adherence to a GFD may lead to a lower BMI in CD due to malabsorption. It is difficult to objectively assess adherence to a GFD; however, constantly positive anti-transglutaminase antibodies are indicative. Our CD patients had a lower BMI-SDS compared with the T1D only group, which is in line with previous reports [2]. Eating disorders, however, were not more frequently observed in CD patients in our data. In contrast, patients with CD are reported to have a higher BMI due to potentially higher fat and caloric uptake [40] or due to adolescents possibly choosing quickly available gluten-free snacks instead of healthy alternatives [41].

The lower BMI-SDS in T1D + CD patients (without depression) could also hint at healthier eating habits, although glycaemic control was not significantly better compared with the T1D only group. Patients with T1D + depression (with or without CD), on the other hand, had worse glycaemic control compared with T1D only, also resulting in higher rates of severe hypoglycaemia and DKA for patients with T1D + depression. Depression in T1D is known to affect adherence to insulin treatment, leading to worse metabolic outcome, higher rates of glycaemic decompensation, and more hospital admissions [24]. ISPAD guidelines also suggest investigating possible eating disorders in T1D patients with worse metabolic control and a multidisciplinary team with a specialist paediatric dietician [3].

Higher frequencies of anxiety and/or depression in adolescent T1D patients are well established. In our study, depression was diagnosed overall in only 2.5%, which is far less than the previously reported 12–30% of adolescents and young adults [23, 25, 42]. Therefore, one might conclude that depression is less frequent in younger T1D patients, which we included in this study. On the other hand, routine depression screening in diabetes centres is not (yet) established and patients with depression might be missed if only clinically diagnosed patients are recorded. This could be a reason for the lower frequency of depression in our cohort.

Patients with depression were older compared with the reference group. Depression might require a certain stage of mental development to occur or to allow diagnosis, as diagnosing depression in young children is difficult [43]. As reported before, CD patients were younger compared with the reference group, implying a greater risk of CD in patients with earlier T1D onset [2]. Early screening for CD and depression in T1D patients may help improve quality of life and prevent consequences of undetected comorbidity.

As stated earlier, CD has been reported to be associated with a number of neurological and psychiatric comorbidities such as ADHD, autism, anxiety, and schizophrenia [12]. Interestingly, in our cohort none of these comorbidities were significantly more frequent in CD patients compared with the T1D only group. However, all of these were significantly more frequent in patients with depression (with or without additional CD). Depression is known to be associated with other psychiatric comorbidities and even with conditions such as autism [44].

In our registry, CD was diagnosed in 4.4% of patients, either via biopsy or via clinical diagnosis, which is in line with previous reports [2, 45]. 62.2% of our CD patients had biopsy-proven CD. According to several guidelines [1, 17], among other autoimmune diseases, paediatric patients with T1D are recommended to be screened for CD autoantibodies, since links between T1D and CD are well known [2] and attributed to their common HLA haplotype DR3-DQ2 or DR4-DQ8 [46]. Until recently, guidelines recommended duodenal biopsies at multiple sites to confirm CD in asymptomatic patients [8, 17].

There is still no definitive statement on whether there is an association between sex and CD [2]. However, female sex is widely known to be associated with autoimmune diseases in general, though for yet unknown reasons [47]. Our findings imply an association of female sex and both CD and depression. Charles et al. suggested a 2:1 ratio of females to males concerning mental health disorders [43], which is similar in our patients with CD and depression (65.2% female). Therefore, we suggest that especially female patients should be screened for CD or signs of depression.

Major strengths of this study include the big sample size of paediatric T1D patients from different European countries and the representative nature of the DPV registry for young T1D patients in Germany, Austria, and Luxemburg. One weakness that is inherent to the investigated question is the dependence on observational studies; therefore, causality between CD and depression cannot be proved. We are also unable to make statements about the prevalence of depression and CD in the registered patients due to possible underreporting. Validated questionnaires on depression such as the PHQ-9 were not available for our analysis. We do not have information on adherence to a GFD, so we could not analyse possible differences in outcomes between patients adhering to a GFD and patients that did not. Since ethnicity is not a suitable concept in Germany, we used migration background as a covariate instead. Finally, since DPV is a diabetes registry we do not have data on patients with CD but without T1D to analyse the incidence of depression in CD patients compared to healthy controls. However, a recent study found an anxiety prevalence of 62.7% and depression prevalence of 34.9% in CD patients [48].

## Conclusion

Depression is disproportionately more frequent in young T1D patients with additional CD. Aside from the recommended CD screening at T1D diagnosis and in regular intervals thereafter, as suggested by several guidelines, depression screening can be helpful in routine care, especially in patients with diagnosed CD. Interdisciplinary cooperation between diabetologists, psychologists, and gastroenterologists should be promoted.

## Supplementary Information

Supplementary file1 (DOCX 31 kb)

## Abbreviations

CD: Coeliac disease
DKA: Diabetic ketoacidosis
DPV: Diabetes patient follow-up
SDS: Standard deviation score
T1D: Type 1 diabetes
