# Supplementary material for: Coeliac disease is associated with depression in children and young adults with type 1 diabetes: results from a multicentre diabetes registry
Source: Acta Diabetol. 2021 Jan 23;58(5):623–31. doi: 10.1007/s00592-020-01649-8 (PMC8076130; doi:10.1007/s00592-020-01649-8)
Supplement: Supplementary file 1 — Supplementary file1 (DOCX 31 kb) [file 592_2020_1649_MOESM1_ESM.docx]

**Supplement**

**Participating centres**

Aachen - Innere RWTH, Aachen - Uni-Kinderklinik RWTH, Aalen Kinderklinik, Ahlen St. Franziskus Kinderklinik, Aidlingen Praxisgemeinschaft, Altötting Kinderklinik Zentrum Inn-Salzach, Altötting Zentrum Inn-Salzach, Altötting-Burghausen Innere Medizin, Amberg Kinderklinik St. Marien, Amstetten Klinikum Mostviertel Kinderklinik, Arnsberg-Hüsten Karolinenhosp. Kinderabteilung, Asbach Kamillus-Klinik Innere, Aue Helios Kinderklink, Augsburg IV. Med. Uni-Klinik, Augsburg Josefinum Kinderklinik, Augsburg Uni-Kinderklinik, Aurich Kinderklinik, Bad Aibling Internist. Praxis, Bad Aibling Internist. Praxis-2, Bad Hersfeld Innere, Bad Hersfeld Kinderklinik, Bad Kreuznach Diakonie Kikli, Bad Kreuznach-St.Marienwörth-Innere, Bad Kreuznach-Viktoriastift, Bad Kösen Median Kinderklinik, Bad Lauterberg Diabeteszentrum Innere, Bad Mergentheim - Diabetesfachklinik, Bad Mergentheim - Kinderdiabetologische Praxis, Bad Oeynhausen Herz-und Diabeteszentrum NRW, Bad Orb Spessart Klinik, Bad Orb Spessart Klinik Reha, Bad Reichenhall Kreisklinik Innere Med., Bad Salzungen Kinderklinik, Bad Säckingen Hochrheinklinik Innere, Bad Waldsee Kinderarztpraxis, Basel Uni-Kinderspital beider Basel (UKBB), Bautzen Oberlausitz KK, Bayreuth Innere Medizin, Berchtesgaden CJD, Berchtesgaden CJD-Beruf.REHA, Berchtesgaden MVZ Innere Med, Bergen Gemeinschaftspraxis, Berlin DRK-Kliniken Mitte Innere, Berlin DRK-Kliniken Pädiatrie, Berlin Endokrinologikum, Berlin Evang. Krankenhaus Königin Elisabeth, Berlin Klinik St. Hedwig Innere, Berlin Lichtenberg - Kinderklinik, Berlin Oskar Zieten Krankenhaus Innere, Berlin Schlosspark-Klinik Innere, Berlin St. Josephskrankenhaus Innere, Berlin Virchow-Kinderklinik, Berlin Vivantes Hellersdorf Innere, Bern Inselspital Kinderklinik, Bern Universitätsklinik für Diabetologie und Endokrinologie, Bielefeld Kinderarztpraxis, Bielefeld Kinderklinik Gilead, Bocholt Kinderklinik, Bochum Universitätskinderklinik St. Josef, Bonn Schwerpunktpraxis, Bonn Uni-Kinderklinik, Bottrop Kinderklinik, Bottrop Knappschaftskrankenhaus Innere, Braunfels-Wetzlar Innere, Braunschweig Kinderarztpraxis, Bremen - Kinderklinik Nord, Bremen - Mitte Innere, Bremen Zentralkrankenhaus Kinderklinik, Bremerhaven Kinderklinik, Bruchweiler Edelsteinklinik Kinder-Reha, Böblingen Kinderklinik, Castrop-Rauxel Evangelisches Krankenhaus, Castrop-Rauxel Rochus-Hospital, Celle Klinik für Kinder- und Jugendmedizin, Chemnitz Kinderklinik, Chemnitz-Hartmannsdorf Innere Medizin - DIAKOMED-1, Coburg Innere Medizin, Coburg Kinderklinik, Coesfeld Kinderklinik, Coesfeld/Dülmen Innere Med., Darmstadt Innere Medizin, Darmstadt Kinderklinik Prinz. Margaret, Datteln Vestische Kinderklinik, Deggendorf Gemeinschaftspraxis, Deggendorf Kinderklinik, Deggendorf Medizinische Klinik II, Deggendorf Pädiatrie-Praxis, Delmenhorst Kinderklinik, Dessau Kinderklinik, Detmold Kinderklinik, Dinslaken Kinderklinik, Dornbirn Innere Medizin, Dornbirn Kinderklinik, Dortmund Kinderklinik, Dortmund Knappschaftskrankenhaus Innere, Dortmund Medizinische Kliniken Nord, Dortmund-Hombruch Marienhospital, Dortmund-St. Josefshospital Innere, Dortmund-West Innere, Dresden Neustadt Kinderklinik, Dresden Uni-Kinderklinik, Duisburg Evang. und Johanniter Krhs Innere, Duisburg Malteser Rhein-Ruhr St. Anna Innere, Duisburg Malteser St. Johannes, Duisburg Sana Kinderklinik, Duisburg-Huckingen, Duisburg-Huckingen Malteser Rhein-Ruhr ST. Johannes, Duisburg-St.Johannes Helios, Düren-Birkesdorf Kinderklinik, Düsseldorf Uni-Kinderklinik, Eberswalde Klinikum Barnim Werner Forßmann - Innere, Eckernförde Gem.-Prax, Eisleben Lutherstadt Helios-Klinik, Erfurt Kinderklinik, Erlangen Uni Innere Medizin, Erlangen Uni-Kinderklinik, Essen Diabetes-SPP, Essen Diabetes-Schwerpunktpraxis, Essen Elisabeth Kinderklinik, Essen Kinderarztpraxis, Essen Uni-Kinderklinik, Esslingen Klinik für Kinder und Jugendliche, Eutin Kinderklinik, Eutin St.-Elisabeth Innere, Feldkirch Kinderklinik, Filderstadt Kinderklinik, Flensburg Diakonissen Kinderklinik, Forchheim Diabeteszentrum SPP, Frankenthal Kinderarztpraxis, Frankfurt Diabeteszentrum Rhein-Main-Erwachsenendiabetologie (Bürgerhospital), Frankfurt Diabeteszentrum Rhein-Main-pädiat. Diabetologie (Clementine-Hospital), Frankfurt Uni-Kinderklinik, Frankfurt Uni-Klinik Innere, Frankfurt Uni-Klinik Innere2, Frankfurt-Sachsenhausen Innere, Frankfurt-Sachsenhausen Innere MVZ, Freiburg Kinder-MVZ, Freiburg St. Josef Kinderklinik, Freiburg Uni Innere, Freiburg Uni-Kinderklinik, Freudenstadt Kinderklinik, Friedberg Innere Klinik, Friedrichshafen Kinderklinik, Fulda Innere Medizin, Fulda Kinderklinik, Fürth Kinderklinik, Gaissach Fachklinik der Deutschen Rentenversicherung Bayern Süd, Garmisch-Partenkirchen Kinderklinik, Geislingen Klinik Helfenstein Innere, Gelnhausen Innere, Gelnhausen Kinderklinik, Gelsenkirchen Kinderklinik Marienhospital, Gera Kinderklinik, Gießen Ev. Krankenhaus Mittelhessen, Gießen Uni-Kinderklinik, Graz Uni Innere, Graz Uni-Kinderklinik, Greifswald Uni-Kinderklinik, Gummersbach Oberbergklinikum, Göppingen Innere Medizin, Göppingen Kinderklinik am Eichert, Görlitz Städtische Kinderklinik, Göttingen Uni Gastroenterologie, Göttingen Uni-Kinderklinik, Güstrow Innere, Hachenburg Kinderpraxis, Hagen Kinderklinik, Halberstadt Innere Med. AMEOS Klinik, Halberstadt Kinderklinik AMEOS, Halle Uni-Kinderklinik, Halle-Dölau Städtische Kinderklinik, Hamburg Altonaer Kinderklinik, Hamburg Endokrinologikum, Hamburg Kinderklinik Wilhelmstift, Hamburg-Nord Kinder-MVZ, Hameln Kinderklinik, Hamm Kinderklinik, Hanau Kinderklinik, Hanau St. Vincenz - Innere, Hanau diabetol. Schwerpunktpraxis, Hannover DM-SPP, Hannover Henriettenstift - Innere, Hannover Kinderklinik MHH, Hannover Kinderklinik auf der Bult, Haren Kinderarztpraxis, Heide Kinderklinik, Heidelberg St. Josefskrankenhaus, Heidelberg Uni-Kinderklinik, Heidelberg Uniklinik Innere, Heidenheim Kinderklinik, Heilbronn Innere Klinik, Heilbronn Kinderklinik, Herdecke Kinderklinik, Herford Innere Med I, Herford Kinderarztpraxis, Herford Klinikum Kinder & Jugendliche, Heringsdorf Inselklinik, Hermeskeil Kinderpraxis, Herne Evan. Krankenhaus Innere, Herten St. Elisabeth Innere Medizin, Herzberg Kreiskrankenhaus Innere, Hildburghausen Hennebergklinik, Hildesheim Bernward Krks Kinderheilkunde, Hildesheim GmbH - Innere, Hildesheim Kinderarztpraxis, Hildesheim Kinderklinik, Hinrichsegen-Bruckmühl Diabetikerjugendhaus, Hof Kinderklinik, Hohenmölsen Diabeteszentrum, Homburg Uni-Kinderklinik Saarland, Idar Oberstein Innere, Idar Oberstein Schwerpunktpraxis, Ingolstadt Klinikum Innere, Innsbruck Uni-Kinderklinik, Innsbruck Universitätsklinik Innere, Iserlohn Innere Medizin, Itzehoe Kinderklinik, Jena Kinderarztpraxis, Jena Uni-Kinderklinik, Kaiserslautern Kinderarztpraxis, Kaiserslautern-Westpfalzklinikum Kinderklinik, Kamen Klinikum Westfalen Hellmig Krankenhaus, Kamen MKK - Medizinisches Kompetenzkollegium, Karlsburg Klinik für Diabetes & Stoffwechsel, Karlsruhe Schwerpunktpraxis, Karlsruhe Städtische Kinderklinik, Kassel Klinikum Kinder- und Jugendmedizin, Kassel Städtische Kinderklinik, Kaufbeuren Innere Medizin, Kaufbeuren Kinderklinik, Kempen Heilig Geist - Innere, Kempen Heilig Geist-KHS - Innere, Kempten Oberallgäu Kinderklinik, Kiel Städtische Kinderklinik, Kiel Universitäts-Kinderklinik, Kirchen DRK Krankenhaus Kinderklinik, Kirchheim-Nürtingen Innere, Klagenfurt Innere Med I, Klagenfurt Kinderklinik, Kleve Innere Medizin, Koblenz Kemperhof 1. Med. Klinik, Koblenz Kinderklinik Kemperhof, Konstanz Innere Klinik, Konstanz Kinderklinik, Krefeld Innere Klinik, Krefeld Kinderklinik, Krefeld-Uerdingen St. Josef Innere, Kreischa-Zscheckwitz Klinik Bavaria, Köln Kinderklinik Amsterdamerstrasse, Köln Uni-Kinderklinik, Landau Innere, Landshut Kinderklink, Lappersdorf Kinderarztpraxis, Leer Klinikum - Klinik Kinder & Jugendmedizin, Leipzig Uni-Kinderklinik, Leoben LKH Kinderklinik, Leverkusen Kinderklinik, Lienz BKH Kinderklinik, Lienz Diabetesschwerpunktpraxis für Kinder und Jugendliche, Lilienthal Diabeteszentrum, Limburg Innere Medizin, Lindenfels Luisenkrankenhaus Innere, Lindlar DM-Zentrum, Lingen Kinderklinik St. Bonifatius, Linz KUK MedCampus IV Kinderklinik, Linz Krankenhaus Barmherzige Schwestern Kardiologie Abt. Int. II, Linz Krankenhaus der Barmherzigen Schwestern Kinderklinik, Lippstadt Evangelische Kinderklinik, Ludwigsburg Innere Medizin, Ludwigsburg Kinderklinik, Ludwigshafen Kinderklinik St.Anna-Stift, Ludwigshafen diabetol. SPP, Luxembourg - Centre Hospitalier, Lübeck Uni-Kinderklinik, Lübeck Uni-Klinik Innere Medizin, Lüdenscheid Hilfswerk Kinder & Jugendliche, Lüdenscheid Märkische Kliniken - Kinder & Jugendmedizin, Lünen Klinik am Park, Magdeburg Ki-Klinik St. Marienstift, Magdeburg Städtisches Klinikum Innere, Magdeburg Uni-Kinderklinik, Mainz Uni-Kinderklinik, Manderscheid Rathauspraxis, Mannheim Uni-Kinderklinik, Mannheim Uniklinik Innere Medizin, Marburg - UKGM Endokrinologie & Diabetes, Marburg Uni-Kinderklinik, Marburg Uni-Kinderklinik, Marktredwitz Innere Medizin, Mechernich Kinderklinik, Meissen Kinderklinik Elblandklinikum, Memmingen Internistische Praxis, Memmingen Kinderklinik, Merzig Kinderklinik, Minden Kinderklinik, Moers - St. Josefskrankenhaus Innere, Moers Kinderklinik, Murnau am Staffelsee - diabetol. SPP, Mutterstadt Kinderarztpraxis, Mödling Kinderklinik, Mönchengladbach Kinderklinik Rheydt Elisabethkrankenhaus, Mühlacker Enzkreiskliniken Innere, Mühldorf am Inn Kinderarztpraxis, München 3. Orden Kinderklinik, München Diabetes-Zentrum Süd, München Kinderarztpraxis diabet. SPP, München Praxiszentrum Saarstrasse, München Schwerpunktpraxis, München von Haunersche Kinderklinik, München-Gauting Kinderarztzentrum, München-Harlaching Kinderklinik, München-Schwabing Kinderklinik, Münster Herz Jesu Innere, Münster Ludgerus-Kliniken GmbH, Münster St. Franziskus Kinderklinik, Münster Uni-Kinderklinik, Münster pädiat. Schwerpunktpraxis, Münsterlingen Kinderklinik, Nagold Kreiskrankenhaus Innere, Nauen Havellandklinik, Neuburg Kinderklinik, Neumarkt Innere, Neunkirchen Gemeinschaftspraxis Kinderheilkunde, Neunkirchen Innere Medizin, Neunkirchen Marienhausklinik Kohlhof Kinderklinik, Neuruppin Kinderklinik, Neuss Lukas-Krankenhaus Kinderklinik, Neuss Lukaskrankenhaus Kinderklinik, Neuwied Kinderklinik Elisabeth, Neuwied Marienhaus Klinikum St. Elisabeth Innere, Nürnberg Cnopfsche Kinderklinik, Nürnberg Uniklinik Med. Klinik 4, Nürnberg Uniklinik Zentrum f Neugeb./Kinder & Jugendl., Oberhausen Innere, Oberhausen Kinderklinik, Oberhausen Kinderpraxis, Oberhausen St.Clemens Hospitale Sterkrade, Oberndorf Gastroenterologische Praxis Schwerpunkt Diabetologie, Oberwart - Burgenländische Krankenanstalten Pädiatrie, Offenbach/Main Innere Medizin, Offenbach/Main Kinderklinik, Offenburg Kinderklinik, Oldenburg Kinderklinik, Oldenburg Schwerpunktpraxis Pädiatrie, Olpe pädiatrische Gemeinschaftspraxis, Oschersleben MEDIGREIF Bördekrankenhaus, Osnabrück Christliches Kinderhospital, Osterkappeln Innere, Ottobeuren Kreiskrankenhaus, Oy-Mittelberg Hochgebirgsklinik Kinder-Reha, Paderborn St. Vincenz Kinderklinik, Papenburg Marienkrankenhaus Kinderklinik, Passau Kinderarztpraxis, Passau Kinderklinik, Pforzheim Kinderklinik, Pfullendorf Innere Medizin, Pirmasens Städtisches Krankenhaus Innere, Plauen Vogtlandklinikum, Prenzlau Krankenhaus Innere, Rastatt Gemeinschaftspraxis, Rastatt Kreiskrankenhaus Innere, Ravensburg Kinderklink St. Nikolaus, Recklinghausen Dialysezentrum Innere, Regensburg Kinderklinik St. Hedwig, Remscheid Kinderklinik, Rendsburg Kinderklinik, Reutlingen Kinderarztpraxis, Reutlingen Kinderklinik, Reutlingen Klinikum Steinenberg Innere, Reutte Tirol BKH Kinderklinik, Rheine Mathiasspital Kinderklinik, Ried Innkreis Barmherzige Schwestern, Rodalben St. Elisabeth, Rosenheim Innere Medizin, Rosenheim Kinderklinik, Rosenheim Schwerpunktpraxis, Rostock Uni-Kinderklinik, Rostock Universität Innere Medizin, Rotenburg/Wümme Agaplesion Diakonieklinikum Kinderabteilung, Rüsselsheim Kinderklinik, Rüsselsheim MVZ, Saaldorf-Surheim Diabetespraxis, Saalfeld Thüringenklinik Kinderklinik, Saarbrücken Kinderklinik Winterberg, Saarbrücken Kinderklinik Winterberg 2, Saarlouis Kinderklinik, Salzburg Universität Innere Medizin, Salzburg Universitäts-Kinderklinik, Scheibbs Landesklinikum, Scheidegg Prinzregent Luitpold, Scheidegg Reha-Kinderklinik Maximilian, Schleswig Heliosklinik Kinderklinik, Schw. Gmünd Stauferklinik Kinderklinik, Schweinfurt Kinderklinik, Schwerin Innere Medizin, Schwerin Kinderklinik, Schwäbisch Hall Diakonie Innere Medizin, Schwäbisch Hall Diakonie Kinderklinik, Siegen Kinderklinik, Singen - Hegauklinik Kinderklinik, Singen Kinderarztpraxis, Sinsheim Innere, Spaichingen Innere, Speyer Diakonissen Stiftungskrankenhaus Pädiatrie, St. Augustin Kinderklinik, St. Johann Tirol Kinderklinik, St. Pölten Universitäts-Kinderklinik, St. Pölten Universitätsklinik Innere, Stade Kinderklinik, Stockerau Landeskrankenhaus, Stolberg Kinderklinik, Stuttgart Olgahospital Kinderklinik, Suhl Kinderklinik, Sylt Rehaklinik, Tettnang Innere Medizin, Traunstein Kinderklinik, Traunstein diabetol. Schwerpunktpraxis, Trier Kinderklinik der Borromäerinnen, Trostberg Innere, Tübingen Uni-Kinderklinik, Ulm Endokrinologikum, Ulm Endokrinologikum Amedes, Ulm Schwerpunktpraxis Bahnhofsplatz, Ulm Uni Innere Medizin, Ulm Uni-Kinderklinik, Vechta Kinderklinik, Viersen Kinderkrankenhaus St. Nikolaus, Viersen internist. Praxis, Villach Kinderklinik, Villingen-Schwenningen Schwarzwald Baar Klinikum Kinderklinik, Villingen-Schwenningen Schwarzwald-Baar-Klinikum Innere, Vöcklabruck Kinderklinik, Waldshut Kinderpraxis, Waldshut-Tiengen Kinderpraxis Biberbau, Wangen Oberschwabenklinik Innere Medizin, Waren-Müritz Kinderklinik, Weiden Kinderklinik, Weingarten Kinderarztpraxis, Weisswasser Kreiskrankenhaus, Wels Innere, Wels Klinikum Pädiatrie, Wernberg-Köblitz SPP, Werningerode MVZ, Wesel Marienhospital Kinderklinik, Wetzlar Schwerpunkt-Praxis, Wien 3. Med. Hietzing Innere, Wien KH Nord-Klinik Floridsdorf, Wien Preyersches Kinderspital, Wien Rudolfstiftung 1. Med. Abtl., Wien SMZ Ost Donauspital, Wien Uni Innere Med III, Wien Uni-Kinderklinik, Wien Wilhelminenspital 5. Med. Abteilung, Wiesbaden Helios Horst-Schmidt-Kinderkliniken, Wiesbaden Kinderklinik DKD, Wilhelmshaven Kinderarztpraxis, Wilhelmshaven Klinikum Kinderklinik, Wilhelmshaven St. Willehad Innere, Winnenden Rems-Murr Kinderklinik, Wismar Kinderklinik, Witten Kinderarztpraxis, Wittenberg Innere Medizin, Wittenberg Kinderklinik, Wittlich DSP, Wolgast Innere Medizin, Worms - Weierhof, Worms Kinderklinik, Worms Schwerpunktpraxis, Wuppertal Universitäts-Kinderklinik, Würzburg Kinderarztpraxis, Zams Kinderklinik, Zweibrücken Ev. KH. Innere, Zweibrücken Kinderarztpraxis, Zwettl Landesklinikum Gmünd-Waidhofen, Zwettl Landesklinikum Gmünd-Waidhofen Kinderklinik.
